# Supplementary material for: BMI-mediated association between glyphosate exposure and increased risk of atherosclerotic heart disease: A large-scale cross-sectional study
Source: PLoS One. 2025 Jan 24;20(1):e0317908. doi: 10.1371/journal.pone.0317908 (PMC11759382; doi:10.1371/journal.pone.0317908)
Supplement: S1 Table — Q1, 0–25%; Q2, 25%-50%; Q3, 50%-75%; Q4, 75–100%. (DOCX) [file pone.0317908.s001.docx]

**S1 Table.** **Basic Characteristics of Glyphosate-Exposed Population in 2013-2014 (Grouped by Presence of CVD).**

|  | Overall | Non-CVD | CVD | p |
| --- | --- | --- | --- | --- |
| **n** | 1602 | 1433 | 169 |  |
| **Age, n(%)** |  | | | <0.001 |
| 20-40years | 555 (37.7) | 547 (41.1) | 8 (5.7) |  |
| 41-60years | 569 (36.8) | 532 (38.3) | 37 (22.1) |  |
| >60years | 478 (25.5) | 354 (20.6) | 124 (72.2) |  |
| **Sex, n(%)** |  | | | <0.001 |
| Male | 780 (48.4) | 689 (48.1) | 91 (50.8) |  |
| Female | 822 (51.6) | 744 (51.9) | 78 (49.2) |  |
| **Rath, n(%)** |  | | | 0.031 |
| Mexican American | 213 (8.8) | 198 (9.2) | 15 (4.8) |  |
| Other races | 370 (13.8) | 342 (14.1) | 28 (10.6) |  |
| Non-Hispanic White | 723 (66.1) | 627 (65.1) | 96 (75.9) |  |
| Non-Hispanic Black | 296 (11.3) | 266 (11.6) | 30 (8.6) |  |
| **Marriage, n(%)** |  | | | 0.034 |
| Married/living with a partner | 968 (62.8) | 868 (63.0) | 100 (60.6) |  |
| Widowed/Divorced or separated | 344 (18.9) | 290 (18.0) | 54 (27.6) |  |
| Never married | 290 (18.3) | 275 (19.0) | 15 (11.8) |  |
| **Education, n(%)** |  | | | 0.002 |
| ＜High school | 311 (13.9) | 258 (12.9) | 53 (24.0) |  |
| High school | 357 (20.8) | 321 (20.8) | 36 (20.9) |  |
| ＞High school | 934 (65.3) | 854 (66.4) | 80 (55.1) |  |
| **PIR, n(%)** |  | | | 0.001 |
| Low poverty | 316 (14.2) | 274 (14.3) | 42 (13.4) |  |
| Moderate poverty | 707 (38.3) | 621 (36.5) | 86 (54.7) |  |
| Extreme poverty | 579 (47.6) | 538 (49.2) | 41 (31.8) |  |
| **Smoking, n(%)** |  | | | 0.077 |
| Never | 883 (56.1) | 812 (57.5) | 71 (42.3) |  |
| Ever | 382 (24.4) | 323 (23.7) | 59 (30.6) |  |
| Current | 337 (19.5) | 298 (18.7) | 39 (27.2) |  |
| **Alcohol users, n(%)** |  | | | <0.001 |
| Never | 232 (12.8) | 201 (12.6) | 31 (15.2) |  |
| Ever | 265 (14.2) | 209 (12.3) | 56 (32.7) |  |
| Light/moderate | 814 (53.1) | 750 (54.5) | 64 (40.3) |  |
| Heavy | 291 (19.8) | 273 (20.7) | 18 (11.9) |  |
| **Activities, n(%)** |  | | | 0.009 |
| inactivist | 785 (46.4) | 663 (44.4) | 122 (64.7) |  |
| activists | 817 (53.6) | 770 (55.6) | 47 (35.3) |  |
| **BMI, n(%)** |  | | | 0.065 |
| ≤25 kg/m2 | 481 (29.8) | 443 (30.5) | 38 (22.4) |  |
| 25–30 kg/m2 | 523 (31.9) | 468 (32.0) | 55 (30.8) |  |
| >30 kg/m2 | 598 (38.3) | 522 (37.4) | 76 (46.9) |  |
| **Hyperlipidemia, n(%)** |  | | | <0.001 |
| No | 494 (30.7) | 472 (33.0) | 22 (9.9) |  |
| Yes | 1108 (69.3) | 961 (67.0) | 147 (90.1) |  |
| **Hypertension, n(%)** |  | | | <0.001 |
| No | 754 (50.5) | 731 (54.1) | 23 (16.3) |  |
| Yes | 848 (49.5) | 702 (45.9) | 146 (83.7) |  |
| **Diabetes, n(%)** |  |  |  | <0.001 |
| No | 1328 (86.2) | 1229 (88.5) | 99 (64.5) |  |
| Yes | 274 (13.8) | 204 (11.5) | 70 (35.5) |  |
| **Congestive Heart Failure, n(%)** |  | | | <0.001 |
| No | 1544 (97.1) | 1433 (100.0) | 111 (70.2) |  |
| Yes | 58 (2.9) | 0 (0.0) | 58 (29.8) |  |
| **Coronary Heart Disease, n(%)** |  |  |  | <0.001 |
| No | 1525 (95.4) | 1433 (100.0) | 79 (52.5) |  |
| Yes | 77 (4.6) | 0 (0.0) | 92 (47.5) |  |
| **Angina, n(%)** |  |  |  | <0.001 |
| No | 1560 (97.7) | 1433 (100.0) | 127 (76.5) |  |
| Yes | 42 (2.3) | 0 (0.0) | 42 (23.5) |  |
| **Heart Attack, n(%)** |  |  |  | <0.001 |
| No | 1536 (96.7) | 1433 (100.0) | 103 (66.1) |  |
| Yes | 66 (3.3) | 0 (0.0) | 66 (33.9) |  |
| **Stroke, n(%)** |  |  |  | <0.001 |
| No | 1553 (97.2) | 1433 (100.0) | 120 (70.4) |  |
| Yes | 49 (2.8) | 0 (0.0) | 49 (29.6) |  |
| **Glyphosate, class (%)** |  |  |  | 0.012 |
| Q1 | 402 (25.4) | 372 (26.3) | 30 (16.8) |  |
| Q2 | 399 (25.7) | 361 (26.2) | 38 (21.6) |  |
| Q3 | 398 (25.4) | 359 (25.3) | 39 (26.7) |  |
| Q4 | 403 (23.4) | 341 (22.2) | 62 (34.9) |  |

Q1, 0-25%; Q2, 25%-50%; Q3, 50%-75%; Q4, 75-100%.
