# Supplementary material for: BMI-mediated association between glyphosate exposure and increased risk of atherosclerotic heart disease: A large-scale cross-sectional study
Source: PLoS One. 2025 Jan 24;20(1):e0317908. doi: 10.1371/journal.pone.0317908 (PMC11759382; doi:10.1371/journal.pone.0317908)
Supplement: S2 Table — Q1, 0–25%; Q2, 25%-50%; Q3, 50%-75%; Q4, 75–100%. (DOCX) [file pone.0317908.s002.docx]

**S2 Table. Basic Characteristics of Glyphosate-Exposed Population in 2013-2014 (Grouped by Presence of ASCVD).**

|  | Overall | Non-ASCVD | ASCVD | p |
| --- | --- | --- | --- | --- |
| **n** | 1602 | 1446 | 156 |  |
| **Age, n(%)** |  |  |  | <0.001 |
| 20-40years | 555 (37.7) | 548 (40.7) | 7 (6.0) |  |
| 41-60years | 569 (36.8) | 534 (38.4) | 35 (20.1) |  |
| >60years | 478 (25.5) | 364 (20.9) | 114 (73.9) |  |
| **Sex, n(%)** |  |  |  | 0.319 |
| Male | 780 (48.4) | 693 (47.8) | 87 (54.1) |  |
| Female | 822 (51.6) | 753 (52.2) | 69 (45.9) |  |
| **Rath, n(%)** |  |  |  | 0.041 |
| Mexican American | 213 (8.8) | 200 (9.2) | 13 (4.7) |  |
| Other races | 370 (13.8) | 343 (14.0) | 27 (11.5) |  |
| Non-Hispanic White | 723 (66.1) | 632 (65.2) | 91 (76.1) |  |
| Non-Hispanic Black | 296 (11.3) | 271 (11.7) | 25 (7.6) |  |
| **Marriage, n(%)** |  |  |  | 0.036 |
| Married/living with a partner | 968 (62.8) | 877 (63.0) | 91 (60.8) |  |
| Widowed/Divorced or separated | 344 (18.9) | 293 (18.0) | 51 (28.2) |  |
| Never married | 290 (18.3) | 276 (19.0) | 14 (11.0) |  |
| **Education, n(%)** |  |  |  | 0.002 |
| ＜High school | 311 (13.9) | 262 (13.0) | 49 (24.0) |  |
| High school | 357 (20.8) | 323 (20.7) | 34 (22.0) |  |
| ＞High school | 934 (65.3) | 861 (66.4) | 73 (54.0) |  |
| **PIR, n(%)** |  |  |  | 0.008 |
| Low poverty | 316 (14.2) | 276 (14.2) | 40 (14.1) |  |
| Moderate poverty | 707 (38.3) | 629 (37.0) | 78 (51.8) |  |
| Extreme poverty | 579 (47.6) | 541 (48.8) | 38 (34.0) |  |
| **Smoking, n(%)** |  |  |  | 0.081 |
| Never | 883 (56.1) | 818 (57.4) | 65 (41.8) |  |
| Ever | 382 (24.4) | 327 (23.7) | 55 (31.6) |  |
| Current | 337 (19.5) | 301 (18.9) | 36 (26.6) |  |
| **Alcohol users, n(%)** |  |  |  | <0.001 |
| Never | 232 (12.8) | 204 (12.7) | 28 (13.5) |  |
| Ever | 265 (14.2) | 215 (12.5) | 50 (32.1) |  |
| Light/moderate | 814 (53.1) | 751 (54.0) | 63 (43.9) |  |
| Heavy | 291 (19.8) | 276 (20.7) | 15 (10.4) |  |
| **Activities, n(%)** |  |  |  | 0.016 |
| inactivist | 785 (46.4) | 672 (44.7) | 113 (63.6) |  |
| activists | 817 (53.6) | 774 (55.3) | 43 (36.4) |  |
| **BMI, n(%)** |  |  |  | 0.248 |
| ≤25 kg/m2 | 481 (29.8) | 445 (30.3) | 36 (24.0) |  |
| 25–30 kg/m2 | 523 (31.9) | 471 (31.9) | 52 (31.8) |  |
| >30 kg/m2 | 598 (38.3) | 530 (37.8) | 68 (44.2) |  |
| **Hyperlipidemia, n(%)** |  |  |  | <0.001 |
| No | 494 (30.7) | 474 (32.7) | 20 (9.9) |  |
| Yes | 1108 (69.3) | 972 (67.3) | 136 (90.1) |  |
| **Hypertension, n(%)** |  |  |  | <0.001 |
| No | 754 (50.5) | 732 (53.6) | 22 (17.6) |  |
| Yes | 848 (49.5) | 714 (46.4) | 134 (82.4) |  |
| **Diabetes, n(%)** |  |  |  | <0.001 |
| No | 1328 (86.2) | 1237 (88.2) | 91 (64.8) |  |
| Yes | 274 (13.8) | 209 (11.8) | 65 (35.2) |  |
| **Congestive Heart Failure, n(%)** |  |  |  | <0.001 |
| No | 1544 (97.1) | 1433 (99.1) | 111 (77.0) |  |
| Yes | 58 (2.9) | 13 (0.9) | 45 (23.0) |  |
| **Coronary Heart Disease, n(%)** |  |  |  | <0.001 |
| No | 1525 (95.4) | 1446 (100.0) | 79 (47.9) |  |
| Yes | 77 (4.6) | 0 (0.0) | 77 (52.1) |  |
| **Angina, n(%)** |  |  |  | <0.001 |
| No | 1560 (97.7) | 1446 (100.0) | 114 (74.2) |  |
| Yes | 42 (2.3) | 0 (0.0) | 42 (25.8) |  |
| **Heart Attack, n(%)** |  |  |  | <0.001 |
| No | 1536 (96.7) | 1446 (100.0) | 90 (62.8) |  |
| Yes | 66 (3.3) | 0 (0.0) | 66 (37.2) |  |
| **Stroke, n(%)** |  |  |  | <0.001 |
| No | 1553 (97.2) | 1446 (100.0) | 107 (67.5) |  |
| Yes | 49 (2.8) | 0 (0.0) | 49 (32.5) |  |
| **Glyphosate, class (%)** |  |  |  | 0.011 |
| Q1 | 402 (25.4) | 375 (26.4) | 27 (15.1) |  |
| Q2 | 399 (25.7) | 365 (26.1) | 34 (22.0) |  |
| Q3 | 398 (25.4) | 361 (25.2) | 37 (28.3) |  |
| Q4 | 403 (23.4) | 345 (22.4) | 58 (34.7) |  |

Q1, 0-25%; Q2, 25%-50%; Q3, 50%-75%; Q4, 75-100%.
