# Supplementary material for: BMI-mediated association between glyphosate exposure and increased risk of atherosclerotic heart disease: A large-scale cross-sectional study
Source: PLoS One. 2025 Jan 24;20(1):e0317908. doi: 10.1371/journal.pone.0317908 (PMC11759382; doi:10.1371/journal.pone.0317908)
Supplement: S3 Table — (DOCX) [file pone.0317908.s003.docx]

**S3 Table. Multivariable Linear Regression of Inflammatory Markers, BMI, and Glyphosate Exposure Levels.**

|  | **β** | **95%CI** | **P-value** |
| --- | --- | --- | --- |
| **White blood cells (1000 cells/μL)** | 0.1854 | (-0.1276, 0.4984) | 0.25 |
| **Neutrophils (1000 cells/μL)** | 1.3386 | (-0.0085, 2.6856) | 0.05 |
| **Lymphocytes (1000 cells/μL)** | -0.0153 | (-0.1216, 0.0910) | 0.78 |
| **Alkaline phosphatase (IU/L)** | 3.3299 | (0.0909, 6.5689) | <0.05 |
| **BMI** | 0.7193 | (0.1682, 1.2703) | <0.05 |
