# Supplementary material for: BMI-mediated association between glyphosate exposure and increased risk of atherosclerotic heart disease: A large-scale cross-sectional study
Source: PLoS One. 2025 Jan 24;20(1):e0317908. doi: 10.1371/journal.pone.0317908 (PMC11759382; doi:10.1371/journal.pone.0317908)
Supplement: S4 Table — (DOCX) [file pone.0317908.s004.docx]

**S4 Table. Mediating effects of inflammatory factors and BMI on the association between glyphosate exposure and ASCVD prevalence.**

| **Inflammatory factors** | **Indirect effects** | **Direct effects** | **Total effects** | **Mediated proportion(%)** | **P-value** |
| --- | --- | --- | --- | --- | --- |
|  | **β(95%)** | **β(95%)** | **β(95%)** |  |  |
| **White blood cells** | 0.000271  (-0.001170, 0.00) | 0.043523  (−0.011838, 0.11) | 0.043794  (−0.011754, 0.12) | 0.61% | 0.84 |
| **Neutrophil cells** | 0.001980  (-0.000361, 0.01) | 0.039268  (-0.000492, 0.10) | 0.041249  (0.001866, 0.10) | 4.80% | 0.10 |
| **Lymphocyte cells** | 0.000345  (-0.002237, 0.00) | 0.041454  (0.002166, 0.10) | 0.041799  (0.003346, 0.10) | 0.82% | 0.82 |
| **Alkaline phosphatase** | 0.000110 (−0.002557, 0.00) | 0.045173  (−0.005571, 0.11) | 0.045283  (-0.005564, 0.11) | 0.24% | 0.98 |
| **BMI** | 0.002405  (0.000182, 0.01) | 0.039336  (0.000391, 0.09) | 0.041741  (0.002112, 0.10) | 5.76% | <0.05 |
