# Supplementary material for: BMI-mediated association between glyphosate exposure and increased risk of atherosclerotic heart disease: A large-scale cross-sectional study
Source: PLoS One. 2025 Jan 24;20(1):e0317908. doi: 10.1371/journal.pone.0317908 (PMC11759382; doi:10.1371/journal.pone.0317908)
Supplement: S5 Table — *Q1, 0–25%; Q2, 25%-50%; Q3, 50%-75%; Q4, 75–100%. Model 4: Model 3 + Aspartatetransaminase, Alaninetransaminase, γ-glutamyl transpeptadase. Model 7: Model 3 + Serum creatinine, Urine creatinine, Blood urea nitrogen, Glomerular filtration rate, Urinary albumin creatinine ratio, Uric acid. Model 6: Model 3 + removing the missing values, Q1: 0–25.04%, Q2: 25.04–50%, Q3: 50–75.04%, Q4: 75.04–100%. (DOCX) [file pone.0317908.s005.docx]

**S5 Table. OR(95%CI) of ASCVD according to different level of Glyphosate exposure after further adjustment of several biomarkers.**

|  | Model 4 | | Model 5 | | Model 6 | |
| --- | --- | --- | --- | --- | --- | --- |
|  | OR (95%CI) | P Value | OR (95%CI) | P Value | OR (95%CI) | P Value |
| Q1 | Ref | Ref | Ref | Ref | Ref | Ref |
| Q2 | 1.43(0.84, 2.43) | 0.17 | 1.45(0.74, 2.83) | 0.26 | 1.48(0.69, 3.18) | 0.30 |
| Q3 | 2.12(1.03, 4.38) | <0.05 | 2.52(1.07, 5.93) | <0.05 | 2.19(0.95, 5.07) | 0.06 |
| Q4 | 1.99(1.14, 3.48) | <0.05 | 2.28(1.31, 3.97) | <0.01 | 1.97(0.97, 3.98) | 0.06 |

*Q1, 0-25%; Q2, 25%-50%; Q3, 50%-75%; Q4, 75-100%.

Model 4: Model 3 + Aspartatetransaminase, Alaninetransaminase, [γ-glutamyl transpeptadase](https://suoxie.supfree.net/wang.asp?id=10058);

Model 7: Model 3 + Serum creatinine, Urine creatinine, Blood urea nitrogen, Glomerular filtration rate, Urinary albumin creatinine ratio, Uric acid;

Model 6: Model 3 + removing the missing values, Q1: 0-25.04%, Q2: 25.04-50%, Q3: 50-75.04%, Q4: 75.04-100%;
